# Combining varenicline and nicotine patches: a randomized controlled trial study in smoking cessation

**DOI:** 10.1186/s12916-014-0172-8

**Published:** 2014-10-08

**Authors:** Josep M Ramon, Sergio Morchon, Antoni Baena, Cristina Masuet-Aumatell

**Affiliations:** Bellvitge Biomedical Research Institute (IDIBELL), Smoking Cessation Clinic, Preventive Medicine Department, Bellvitge University Hospital, Feixa Llarga s/n 08907 Hospitalet de Llobregat, Barcelona, Spain; Medical Sciences Department, School of Medicine, Barcelona University, Feixa Llarga s/n 08907 Hospitalet de Llobregat, Barcelona, Spain

**Keywords:** Smoking cessation, Varenicline, Nicotine patches, Combination therapy, Randomized trial

## Abstract

**Background:**

Some smokers may benefit from a therapy that combines different nicotine replacement therapies (NRT) or drugs with different mechanisms of action.

The aim of this study was to determine the efficacy of the combined therapy of varenicline and nicotine patches versus varenicline monotherapy.

**Methods:**

Three hundred forty-one smokers who smoked 20 or more cigarettes per day were recruited from a smoking cessation clinic between February 2012 and June 2013. The participants were randomized to receive a varenicline plus nicotine patch of 21 mg every 24 hours (170) or varenicline plus a placebo patch (171). All of the smokers received a standard 12-week course of varenicline and an 11-week course of either the placebo patch or the active patch after the target quit day. Both groups received behavioral support. The primary outcome was continuous abstinence for weeks 2 through 12 confirmed by exhaled levels of carbon monoxide. *Post hoc* subgroup analyses were performed to evaluate the treatment effects for a specific endpoint in subgroups of smokers.

**Results:**

The combination of the nicotine patch with varenicline was not associated with higher rates of continuous abstinence at 12 weeks (39.1% versus 31.8%; odds ratio (OR) 1.24; 95% confidence interval (CI) 0.8 to 2.6) and 24 weeks (32.8% versus 28.2%; OR 1.17; 95% CI 0.4 to 1.9). When participants were analyzed by subgroups according to cigarette consumption, the abstinence rates among smokers who smoked more than 29 cigarettes per day at 12 weeks (OR 1.39; 95% CI 1.2 to 2.5) and 24 weeks (OR 1.46; 95% CI 1.2 to 2.8) were significantly higher in the combination group. Other *post hoc* analyses based on level of dependence and previous quit attempts did not show subgroup differences. No differences between the groups for the reported adverse events were observed (χ2 value 0.07; *P* 0.79).

**Conclusions:**

The combination of varenicline with the nicotine patch does not improve abstinence rates at 12 and 24 weeks compared with varenicline used as monotherapy when all smokers were analyzed as a whole, independent of consumption level.

**Trial registration:**

This study is registered at clinicaltrial.gov (NCT01538394).

## Background

None of the available first-line pharmacological therapies to treat tobacco dependence have been labelled in Spain for use in combination with other therapies. Seven first-line pharmacotherapies that are currently available are recommended in clinical practice guidelines to treat tobacco dependence; all of these therapies have been tested to be effective for increasing tobacco abstinence rates when used as monotherapies in comparison with a placebo [1]. However, not all smokers are able to quit with monotherapy. Some smokers may benefit from a combination therapy featuring the simultaneous use of different nicotine replacement therapies (NRT) or drugs with different mechanisms of action (for example, NRT and bupropion). In a recent meta-analysis conducted by Mills *et al*. [2], treatment with NRT did not show a superior effect compared with the standard treatment of either nicotine patches or varenicline used as monotherapies. In contrast, in the meta-analysis by Cahill *et al*. [3], varenicline was shown to be superior to both NRT (odds ratio (OR) 1.57; 95% confidence interval (CI) 1.29 to 1.91) and bupropion (OR 1.59; 95% CI 1.29 to 1.96) but not to an NRT combination (OR 1.06; 95% CI 0.75 to 1.48).

Combination therapy with different drugs may provide a therapeutic advantage by increasing the serum nicotine concentrations and capitalizing on the synergy obtained from two different mechanisms of action [4-6].

Therefore, some smokers may benefit from a combination of varenicline and a NRT. Varenicline may not fully saturate the nicotinic receptors, and incompletely saturated receptors may lead to only a partial attenuation of nicotine cravings. If supplemental nicotine replacement therapy is used, more receptor saturation is achieved, which may diminish the urge to smoke more completely.

The evidence from studies with combination therapy using varenicline and NRT is limited. In a study by Ebbert *et al*. [7], a number of pharmacological combination therapies were compared with a standard treatment in a clinical cohort of residential smokers. The study showed that the combination of varenicline and NRT was safe, but no differences were observed from the group that received standard treatment in terms of effectiveness.

Recently, a randomized controlled trial [8] observed that the addition of nicotine patches to varenicline among smokers who smoked 10 or more cigarettes per day did not increase the efficacy of varenicline as a monotherapy. However, a study by Koegelenberg *et al*. [9] suggested that the efficacy of varenicline was enhanced by the addition of nicotine patches.

The aim of this study was to determine the efficacy of combining varenicline with nicotine patches versus varenicline as monotherapy in smokers who smoked twenty or more cigarettes per day for the last six months. The primary end point was the continuous abstinence rate from week two to week twelve of treatment.

## Methods

### Study design

A randomized placebo-controlled clinical trial was conducted at the Smoking Cessation Clinic situated in the University Bellvitge Hospital, Barcelona, Spain, between February 2012 and December 2013.

The center’s ethics committee approved the study. Participants provided written informed consent.

### Participants

The participants were smokers who were seeking treatment in an outpatient smoking cessation clinic between February 2012 and June 2013. The inclusion criteria were as follows: being 18 years old or older, having smoked ≥20 cigarettes daily for the last six months, providing consent to participate and no period of smoking abstinence longer than three months in the last year. Female smokers were eligible provided that they were not breastfeeding, pregnant (negative pregnancy test) or at risk of becoming pregnant. The exclusion criteria were current or past psychotic disorder (schizophrenia), history of suicide attempts, not understanding the Spanish language and current or past alcoholism or other drug addictions.

Smokers who had used nicotine transdermal patches or varenicline (VRN) in the last six months were excluded. Smokers with chronic diseases were not excluded.

After a baseline assessment, the participants were randomly assigned to the treatment arms (varenicline + nicotine patch or varenicline + placebo patch) in a 1:1 ratio. Randomization was performed using a computer-generated randomization system, and random numbers were individually placed into sealed envelopes. An independent study collaborator preserved the sealed envelopes and conducted the allocation, allotting one sealed envelope per patient and opening it in front of the therapist and the patient. The sample size was estimated, accepting Type I error alpha = .05 and 80% power in a two-sided test. Sample size was calculated based on abstinence rates in previous combination NRT and varenicline trials. A total of 170 subjects per group was necessary to detect a statistically significant difference of ≥15.0% between groups.

### Procedures

All participants attended the clinic for baseline assessments one week before the target quit day (TQD). At the baseline assessment visit, written informed consent was obtained, the inclusion and exclusion criteria were reviewed, and the baseline demographic variables and smoking and medical history were collected. Comorbidity and psychosocial characteristics (Beck Depression Inventory [10] and Hamilton Anxiety Rating scales [11] scores and social support) and the Fagerstrom Test for Nicotine Dependence were collected during the baseline interview. All participants started varenicline at the baseline visit and were randomized to receive either the nicotine or placebo patches on the TQD. During the treatment phase, participants visited the clinic at 1, 2, 3, 5, 8, 10, 12 and 24 weeks after the baseline visit.

The Minnesota Nicotine Withdrawal Scale [12], Beck Depression Inventory [10], Hamilton Anxiety Rating Scale [11] and the Scale for Suicide Ideation [13] were recorded at 2, 5, 8, 12 and 24 weeks. At each clinic visit, the use of the trial therapy, adverse events and cigarette consumption since the last clinic visit and the levels of exhaled carbon monoxide were assessed. Weight and blood pressure were determined at each visit. At each visit, smokers participated in behavioral counselling sessions that had been previously standardized. Sessions lasted 10 to 15 minutes each and were based on motivational interviewing. The sessions included practical counselling elements, such as problem solving and skills training.

### Trial medication

Varenicline was used in the standard commercial supply beginning one week before the TQD: 0.5 mg once daily for three days, then 0.5 mg twice daily for four days, followed by 1 mg twice daily for eleven weeks. The smokers received identical packages of either nicotine (Nicotinell® 21 mg/24 hours) or placebo patches for 11 weeks.

### Measures

The primary end point was continuous abstinence defined as not smoking throughout the follow-up period from week 2 (1 week after the quit date) to week 12 [14,15]. The criteria for determining continuous abstinence were not having smoked since week 2 and showing CO concentrations of <10 ppm at 12 weeks. Based on recommendations from a number of authors, subjects who failed to provide validation data were considered relapsed [15,16].

The secondary end points were point prevalence, defined as abstinence during the week before the follow-up visits at 8, 12 and 24 weeks, the continuous abstinence rate from week 2 through 24, and the incidence of adverse events.

All adverse events were recorded at each visit after randomization.

### Statistical analysis

The statistical analysis was performed on the basis of intention to treat. All randomized smokers were included in the analysis. The rates were estimated by including all of the smokers who were allocated into the specific group in the denominator.

The differences in the percentages were assessed using the chi-square test, and the means were compared using a two-sample T-test. A logistic regression model was used to test the efficacy across the different arms of the study. The model was adjusted for potential confounding factors, including gender, age and therapist. The multivariate model included gender and age as covariates because they have been [17] related to increased risk of relapse.

Although no differences were observed between different therapists for the 12-week abstinence rates (33.9%, 36.8%, 35.6%; χ2 value 0.049; *P* 0.82), the counsellor can be a source of bias to control for this potential source of variation; thus, the therapist was introduced in the final model.

*Post hoc* subgroup analyses were performed to evaluate the treatment effects for a specific end point in subgroups of smokers defined by baseline smoking, and the interaction effect was assessed.

A repeated-measures analysis of variance (ANOVA) was performed to study the Minnesota Nicotine Withdrawal Scale (MNWS) scores over time and by group, and Student’s T-test was used to compare MNWS differences between the study groups at single points in time.

SPSS version 14 (SPSS Inc., Chicago, IL, USA) was used for the analyses.

## Results

### Attrition

**Figure 1:**
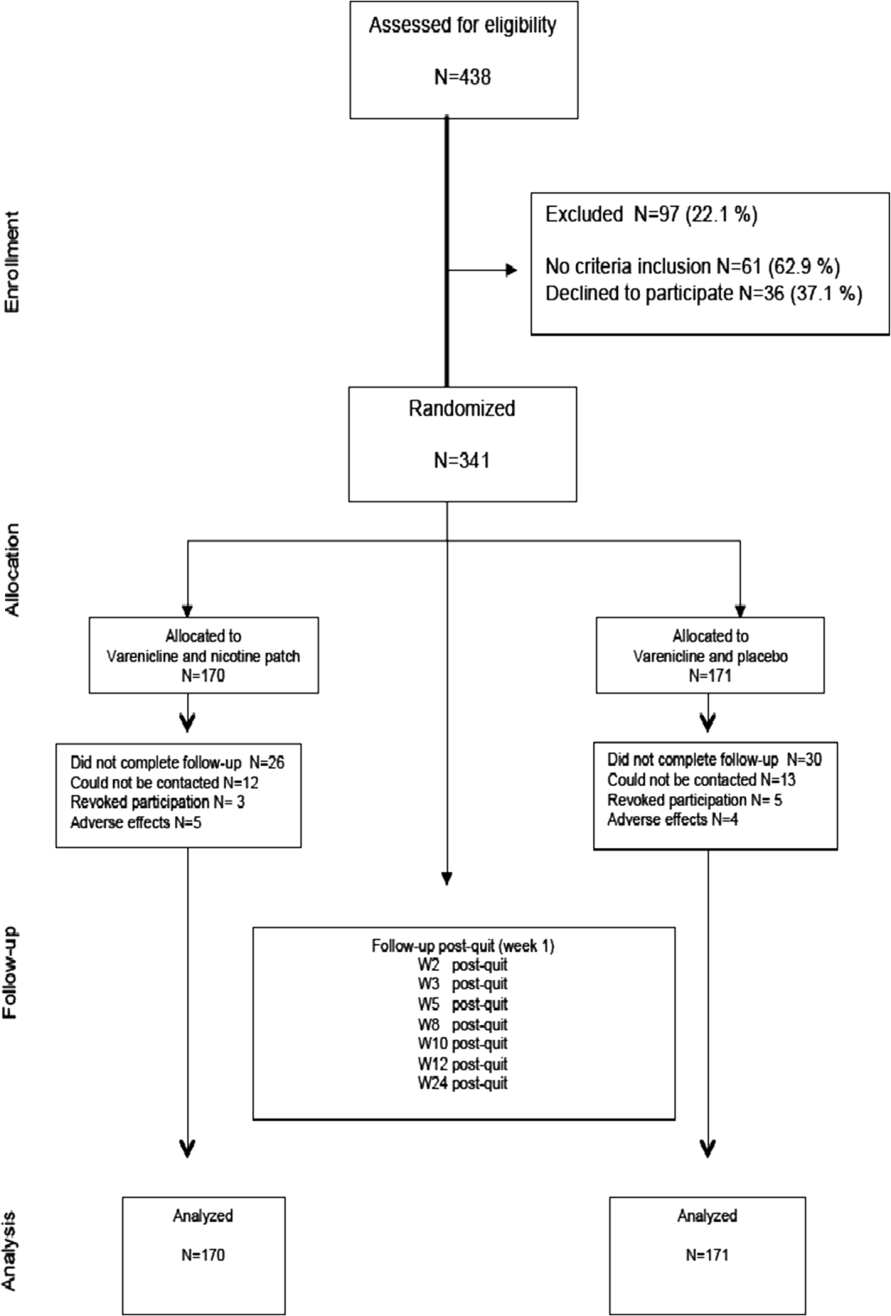
**Flowchart and follow-up.**

Figure 1 shows the flowchart for evaluating the study participants. Of the 438 eligible smokers, 341 (78%) were included and randomly allocated. Sixty-one smokers did not meet the inclusion criteria, and 36 smokers refused to participate. Finally, 170 smokers were assigned to the varenicline and nicotine patch group, and 171 smokers were assigned to the varenicline plus placebo patch group. A total of 243 smokers (71.3%) completed follow-up.

The smokers in the study groups were similar at baseline, and there were no significant differences between the two arms (Table 1). The average daily consumption of cigarettes was 29.2 in the intervention group and 28.7 for the control group (*P* 0.8).

**Table 1:** **Baseline characteristics by group**

**Variables**	**Varenicline + nicotine patch N = 170**	**Varenicline + placebo N = 171**	***P*** **-value**
Gender Number (%)			
Male	95 (55.9%)	102 (59.6%)	
Female	75 (44.1%)	69 (40.4%)	0.55^a^
Age			
Mean (±SD)	44.1 (±14.8)	46.2 (±13.1)	0.38^b^
Cigarettes/day Number (%)			
≤29 cig/day	78 (45.9%)	84 (49.1%)	
>29 cig/day	92 (54.1%)	87 (50.9%)	0.60^a^
Previous attempts Number (%)			
None	33 (19.4%)	27 (15.8%)	
1	73 (42.9%)	67 (39.2%)	
2 to 3	44 (25.9%)	60 (35.1%)	
>3	20 (11.8%)	17 (9.9%)	0.31^a^
FTND			
Mean (±SD)	6.1 (±1.6)	6.8 (±1.8)	0.9^b^

^a^χ2 squared test; ^b^T-test. FTND, Fagerstrom test for nicotine dependence; SD Standard deviation.

### Primary and secondary end points

Table 2 shows both the continuous and point abstinence rates. A comparison of the intervention and control groups for the continuous abstinence rates at 8, 12 and 24 weeks (Table 2) revealed no statistically significant differences (OR 1.04, 95% CI 0.4 to 2.1; OR 1.24, 95% CI 0.8 to 2.6; and OR 1.17 95% CI 0.4 to 1.9, respectively). Additionally, when the groups were compared for point abstinence, the results were similar and not statistically significant (Table 2).

**Table 2:** **Smoking abstinence by group**

**Group**	**Continuous abstinence** ^**a**^			**Seven-day point-prevalence abstinence**		
	**Abstainers (%)**	**Crude OR (95% CI)**	**OR** ^**b**^ **(95% CI)**	**Abstainers (%)**	**Crude OR (95% CI)**	**OR** ^**b**^ **(95% CI)**
Week 8						
Varenicline + nicotine patch (N = 170)	72 (42.2%)	1.08 (0.7 to 1.7)	1.04 (0.4 to 2.1)	80 (47.2%)	1.06 (0.7 to 1.6)	1.02 (0.3 to 1.6)
Varenicline + placebo (N = 171)	69 (40.4%)	1	1	78 (45.7%)	1	1
Week 12						
Varenicline + nicotine patch (N = 170)	66 (39.1%)	1.37 (0.8 to 21.)	1.24 (0.8 to 2.6)	68 (40.2%)	1.37 (0.8 to 2.1)	1.20 (0.7 to 2.1)
Varenicline + placebo (N = 171)	54 (31.8%)	1	1	56 (38.5%)	1	1
Week 24						
Varenicline + nicotine patch (N = 170)	56 (32.8%)	1.25 (0.8 to 2.0)	1.17 (0.4 to 1.9)	60 (35.1%)	1.28 (0.8 to 2.0)	1.15 (0.4 to 2.0)
Varenicline + placebo (N = 171)	48 (28.2%)	1	1	51 (33.4%)	1	1

^a^Continuous abstinence from weeks 2 to 8, 12 and 24 weeks; ^b^adjusted by age, gender and therapist. CI, confidence interval; N, number; OR, odds ratio.

A *post hoc* exploratory analysis was conducted to assess if the effect of treatment was related to cigarette consumption (≤29 cigarettes per day (cpd) versus >29 cpd); nicotine dependence (Fagerstrom test for nicotine dependence (FTND) score ≤6 versus >6); and previous attempts (none, 1 to 3 and more than 3).

The *post hoc* subgroup analyses revealed that the effect of treatment at 12 and 24 weeks was dependent on cigarette consumption at baseline (interaction *P* = 0.02 at 12 weeks and *P* = 0.02 at 24 weeks). A non-significant interaction was detected among participants by the level of nicotine dependence (*P* = 0.06 at 12 weeks and *P* = 0.1 at 24 weeks) and previous attempts (*P* = 0.4 at 12 weeks and *P* = 0.6 at 24 weeks).

Analysis between the subgroups was performed based on the two groups of cigarette smokers, and the rates of continuous abstinence were significantly higher for the combined treatment group than for the control group at week 12 (OR 1.39; 95% CI 1.2 to 2.5) and at week 24 (OR 1.46; 95% CI 1.2 to 2.8) in the subgroup who smoked more than 29 cpd (Table 3). In contrast, the differences in the rates among smokers of 29 or fewer cpd were not significant at weeks 8, 12 and 24.

**Table 3:** **Smoking abstinence by group and cigarette consumption**

**Group**	**Smokers ≤ 29 cigarettes/day**			**Smokers > 29 cigarettes/day**		
**Continuous abstinence**	**Abstainers/N (%)**	**Crude OR (95% CI)**	**OR** ^**a**^ **(95% CI)**	**Abstainers/N (%)**	**Crude OR (95% CI)**	**OR** ^**a**^ **(95% CI)**
Weeks 2 to 8						
Varenicline + nicotine patch	38/78 (48.7%)	1.05 (0.7 to 1.4)	1.0 (0.5 to 1.3)	34/92 (36.9%)	1.13 (0.7 to 1.6)	1.07 (0.6 to 1.8)
Varenicline + placebo	39/84 (46.4%)	1	1	29/87 (33.3%)	1	1
Weeks 2 to 12						
Varenicline + nicotine patch	35/78 (43.6%)	1.14 (0.7 to 1.6)	1.0 (0.5 to 1.8)	31/92 (34.8%)	1.44 (0.9 to 2.3)	1.39 (1.2 to 2.5)
Varenicline + placebo	33/84 (39.2%)	1	1	21/87 (24.1%)	1	1
Weeks 2 to 24						
Varenicline + nicotine patch	27/78 (34.6%)	0.99 (0.6 to 1.5)	1.0 (0.7 to 1.6)	29/92 (31.5%)	1.52 (1.0 to 2.5)	1.46 (1.2 to 2.8)
Varenicline + placebo	30/84 (35.7%)	1	1	18/87 (20.6%)	1	1

^a^Adjusted by age, gender and therapist. CI, confidence interval; N, number; OR, odds ratio.

### Nicotine withdrawal

**Figure 2:**
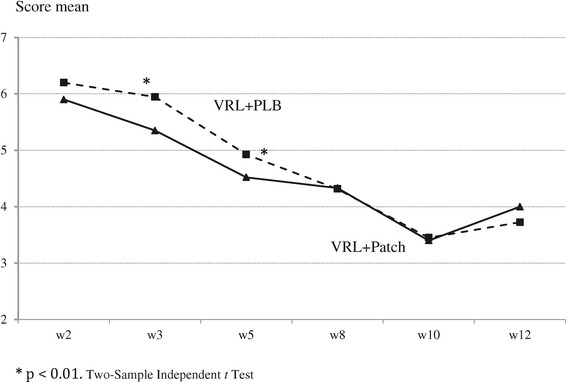
**Minnesota nicotine withdrawal scale by intervention group.**

**Figure 3:**
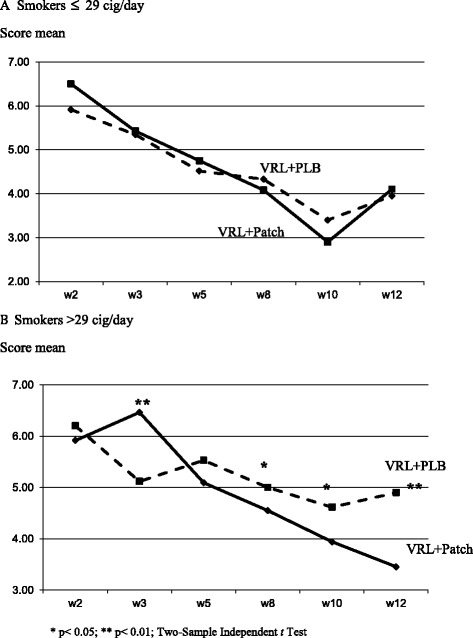
**Minnesota nicotine withdrawal scale by consumption level.** One legend for both panels **A** and **B**. Under-figures: VRL + PLB: Varenicline + Placebo; VRL + Patch: Varenicline + Nicotine Patches.

When comparing the mean scores of the MNWS between the study groups at week 12, the intervention group showed lower withdrawal than did the control group (4.6 versus 4.8, F = 1.346; *P* = 0.05) (Figure 2). The decrease in MNWS scores in both groups based on consumption level (Figures 3A and B) during the follow-up period was statistically significant among smokers who smoked 29 or fewer cpd (*P* <0.01 in the intervention group and *P* <0.05 in the control group) and among smokers of more than 29 cpd (*P* <0.01 in the intervention group and *P* 0.04 in the control group), as assessed by repeated-measures ANOVA.

However, when the scores were compared between the two groups by the cigarette consumption subgroups (Figure 3A and B), no differences were observed at each time point among the smokers with a cigarette consumption ≤29 (Figure 3A) compared with the group of heavy smokers in which the scores were significantly lower at 3, 8, 10 and 12 weeks among those who received the combination therapy (Figure 3B).

### Adverse events

Adverse events occurred in 41.3% of smokers in the combination group compared with 39.7% in the control group (χ2 value 0.07; *P* 0.79). The various adverse events that were observed are shown in Table 4. Insomnia (χ2 value 0.85; *P* 0.35), abnormal dreams (χ2 value 0.21; *P* 0.64) and nausea (χ2 value 0.02; *P* 0.88) were the most frequently reported events in both groups. Headache was more frequently observed in the nicotine patch group (4.1% versus 2.6), but the differences were not statistically significant when both groups were compared (χ2 value 0.86; *P* 0.35). No serious adverse events occurred during follow-up.

**Table 4:** **Adverse events**

**Adverse event**	**Varenicline + Nicotine patch**	**Varenicline + Placebo**
	**Number = 170**	**Number = 171**
	**n (%)**	**n (%)**
Insomnia	29 (17.3%)	23 (13.2%)
Nausea	31 (18.3%)	33 (19.1%)
Abnormal dreams	29 (17.4%)	26 (15.1%)
Constipation	15 (8.8%)	13 (7,6%)
Dyspepsia	10 (5.9%)	8 (4.7%)
Headache	7 (4.1%)	4 (2.6%)
Other^a^	9 (5.3%)	11 (6.4%)

^a^Irritability, depressive symptoms, fatigue, hypotension.

Five smokers in the combination group discontinued treatment because of adverse events (three because of nausea and two because of insomnia), and four smokers in the control group discontinued (one because of depressive symptoms, one because of insomnia and two because of nausea).

No differences between the two groups in relation to medication adherence throughout follow-up were observed. One participant did not use the patch in the intervention group, and two from the control group did not use the patch. All participants in both the intervention and the control groups used varenicline in the first four weeks of treatment. One person in the intervention group but none in the control group failed to use varenicline between weeks 4 and 12, and three in the intervention group and two in the placebo group failed to do so at weeks 8 and 12.

## Discussion

The aim of the present study was to assess the efficacy of the combined use of varenicline and nicotine patches compared with varenicline alone in smoking cessation.

In this study, the observed results showed that the combination of varenicline and the nicotine patch was not associated with an enhanced continuous abstinence rate at 12 and 24 weeks, as well as with the point prevalence abstinence rate at 24 weeks.

The combined use of the nicotine patch and varenicline compared with varenicline alone resulted in higher rates of abstinence at 12 and 24 weeks, but only among smokers of more than 29 cpd.

There are only three studies in the previous literature that measure the efficacy or effectiveness of combining varenicline with nicotine patches. An observational study by Ebbert *et al*. [7] reported no differences in the 30-day point prevalence abstinence rate between patients receiving the usual care and patients treated with varenicline and NRT at the six-month follow-up and in the reported data on the safety and tolerability of the combination of varenicline and NRT. A study by Hajek *et al*. [8], which used the same method as the present trial with medication started as per labelling found no effect of the combination treatment at three months. Koegelenberg *et al*. [9] found the combination more effective than varenicline alone at three and six months, but patch use was started two weeks prior to target quit date. The three studies included smokers of 10 cigarettes or more, in contrast to this study in which the participants were smokers of 20 or more cigarettes. In the Hajek study [8], the continuous abstinence rate at 12 weeks was similar to that observed in this study for all smokers in the combination group as a whole (36% versus 39.1%, respectively), although in those studies, smokers had lower levels of nicotine dependence and more attempts to stop smoking than did the smokers in this study. The abstinence rates were also similar at 12 weeks, which may reflect the existence of other factors such as the size of the study sample. The study by Koegelenberg *et al*. [9] shows higher rates of abstinence than this study, which may be due to the inclusion criteria in relation to the amount of consumption (10 or more versus 20 or more cpd), the possible heterogeneity of the participants and that the nicotine patch was used for two weeks prior to the TQD. The results could reflect the effects of ‘nicotine preloading’ rather than any effects of medication combination.

However, in the sub-analysis of the smokers of 29 or fewer cpd and of those who smoked more than 29 cpd, the abstinence rates at 12 and 24 weeks were higher and more significant among the heavy smokers who had received the combination therapy. These results are similar to those of a study of the combination of bupropion and varenicline for smoking cessation [18].

This study’s sample size was sufficient to detect differences in the abstinence rates equal to or greater than 15% in one of the groups. The dropout rate in both groups was similar, and no significant differences were observed in the participants who withdrew from the study because of adverse events. In this study, the dropouts and the subjects who failed to provide validation data were considered relapsed [15,16], although not all of the smokers who dropped out may have actually relapsed, and an underestimation in the rates could have occurred. However, the number of subjects without information in each group was similar and was not considered to be a potential source of bias.

The different usage periods for both of the medications could represent a possible bias in the results. To control for these biases, the varenicline and patches were administered for 11 weeks. Using 21 mg/24 hours patches in combination therapy did not show an increase in adverse events. The number of patients experiencing headache, insomnia and nausea was similar in both groups and comparable with the rates reported in previous studies using 15 mg/16 hours patches [8,9]. Finally, another potential limitation is the possibility for bias in the non-pharmacological interventions. The study involved three different therapists trained in the standardized study protocol who were experts in smoking cessation. However, and although no differences were observed between different therapists, the counsellor may still have been a source of bias in this study [19]. To control for this potential source of variation, the therapist was introduced in the final model.

## Conclusions

In conclusion, the combination of the varenicline with the nicotine patches does not improve abstinence rates at 12 and 24 weeks compared with varenicline used as monotherapy although the combination treatment may help patients smoking 29 or more cpd. Further research is needed to confirm this *post hoc* finding.
